# A Case-Control Study Measuring Mentalization in Individuals With PTSD Compared to Controls Using the STOMP Task

**DOI:** 10.1192/bjo.2022.211

**Published:** 2022-06-20

**Authors:** Poppy MacInnes, Chantelle Wiseman

**Affiliations:** 1 Royal United Hospital, Bath, United Kingdom.; 2 University of Bristol, Bristol, United Kingdom.; 3 University of Cardiff, Cardiff, United Kingdom

## Abstract

**Aims:**

Social cognition is impaired in a variety of psychiatric conditions; evidence for impairment in individuals with PTSD is increasing. Mentalization is one domain of social cognition that refers to the capacity to understand other people by ascribing mental states to them. The STOMP task (Spontaneous Theory of Mind Protocol) involves an individual watching two minutes of a silent video and describing what they see. As part of a wider project examining social cognition in PTSD, we aimed to find out whether mentalization in the STOMP task differs between patients with PTSD compared to controls.

**Methods:**

171 individuals undertook the task: 30 patients were recruited from centres in Cardiff and Bristol at the start of their psychological therapy; 141 controls were recruited through Prolific website. Participants watched a 2-minute silent video and were asked to write 7–10 sentences about the clip. Qualtrics software selected the video and collected the texts. The verbs of the texts were coded and given a score by PM using the Mental-Physical Verb Norms (MPVN) method. MVPN was developed by Orr et al. 2019, to give a value to 250 commonly used verbs based on their mental or physical attributes (the higher the value, the more ‘mental’ the verb). The total score of each text was divided by the number of verbs scored to produce an average that reflects how much mentalization was used. An unpaired t-test was used to calculate the significance in difference between the means of the two groups.

**Results:**

The overall average score of individuals with PTSD was higher than the controls (38.5 vs. 33.5, p value 0.0047). The median score of individuals with PTSD was 37.95 compared to 31.60 with an actual difference of -6.350 and a Hodges-Lehmann difference of -4.650. These results do not support the hypothesis that mentalization is impaired in patients with PTSD.

**Conclusion:**

This case-control study suggests that mentalization could be enhanced in patients with PTSD compared to controls. These results should be interpreted as part of a wider project being undertaken on the topic of social cognition in PTSD. Further studies with more participants from the population of interest and larger sample sizes could produce more reliable results, together with an expansion of the number of verbs coded in the MPVN method.

